# Enhancing Women’s Financial Empowerment through Savings Strategies: Evidence from MAMIDECOT, Masaka District, Uganda

**DOI:** 10.12688/f1000research.171318.1

**Published:** 2025-11-25

**Authors:** Nakayiso Eseza, Nyambane David Ongabi, Nyakundi Andrew

**Affiliations:** 1 Finance and Accounting, Kampala International University - Western Campus, Bushenyi, Western Region, Uganda; 2 Finance and Accounting, Kampala University School of Business and Management, Kampala, Central Region, Uganda; 3 Finance and Accounting, Kampala University School of Business and Management, Kampala, Central Region, Uganda

**Keywords:** Saving strategies, women’s financial empowerment, Masaka District, Uganda

## Abstract

**Background:**

Women’s financial empowerment is central to achieving inclusive and sustainable development in sub-Saharan Africa. Savings strategies, particularly within Savings and Credit Cooperative Organizations (SACCOs), play a pivotal role in enhancing women’s financial inclusion, independence, and decision-making capacity. Despite progress, rural women in Uganda continue to face barriers such as limited financial literacy, irregular income, and restrictive social norms. This study therefore examined the influence of savings strategies on women’s financial empowerment in Masaka District, Uganda, focusing on structured saving mechanisms and the moderating role of cultural factors.

**Methods:**

A convergent mixed-methods design grounded in the pragmatic paradigm was employed. Quantitative data were collected from 340 women members of MAMIDECOT SACCO through structured questionnaires, while qualitative data were obtained from interviews with 10 SACCO managers and focus group discussions. Quantitative data were analyzed using descriptive statistics, Pearson correlation, and regression analysis in SPSS v25, whereas qualitative data were thematically analyzed to complement statistical results. Reliability (Cronbach’s α ≥ 0.70) and validity were confirmed, and ethical approval was obtained from the Kampala International University Research Ethics Committee.

**Findings:**

Results revealed that savings strategies significantly enhanced women’s financial empowerment (R ^2^ = 0.304, F(4,335) = 36.526, p < 0.001), strengthening financial independence, decision-making, and household influence. Group savings fostered mutual trust, accountability, and self-reliance among members. Cultural factors did not significantly moderate the relationship, indicating that structured savings mechanisms were broadly effective across diverse socio-cultural contexts.

**Conclusions:**

Savings strategies are a critical driver of women’s financial empowerment in rural Uganda. Policymakers and SACCOs should expand gender-sensitive savings products, strengthen governance, and scale up financial literacy programs to sustain empowerment outcomes and promote inclusive economic participation among women.

## 1. Introduction

Women’s financial empowerment is increasingly recognized as a cornerstone of sustainable development, aligning directly with Sustainable Development Goals (SDG) 5 on gender equality and SDG 8 on decent work and economic growth. Savings strategies are widely considered foundational in this process, as they provide mechanisms for resource accumulation, risk management, and long-term financial independence. Evidence suggests that women who adopt structured savings practices gain higher levels of financial literacy, confidence, and bargaining power within households ( Kumar et al., 2020). Ody-Brasier (2017) and Kabandula and Mulenga (2018) similarly demonstrate that savings strengthen women’s decision-making and entrepreneurial capacity, while UN Women (2020) emphasizes the role of savings in enhancing social standing and community participation. Beyond individual and household gains, savings initiatives contribute to resilience against economic shocks and support women’s integration into wider development processes.

Despite these benefits, access to savings mechanisms remains uneven globally, limiting women’s ability to achieve financial empowerment. In Africa, rural women face systemic exclusion from formal financial services, with technological innovations such as mobile money improving inclusion only partially due to digital illiteracy, affordability constraints, and mistrust in financial technology ( Jack & Suri, 2014; Mbiti & Weil, 2016). Community-based savings groups have emerged as alternative pathways, but their effectiveness is inconsistent due to weak governance, irregular contributions, and socio-cultural barriers ( D’Espallier et al., 2017; Kabandula & Mulenga, 2018). In East Africa, rural women continue to confront both institutional and cultural barriers to structured savings, and in Uganda, these challenges are magnified by inadequate financial literacy, poor institutional infrastructure, and entrenched socio-cultural norms ( Mugisha et al., 2020; GSMA, 2023). In Masaka District, the majority of women remain on the periphery of formal savings systems, and empirical research on the drivers, barriers, and outcomes of their participation is scarce. This gap underscores the need for the present study, which seeks to assess the accessibility, utilization, and impact of savings strategies on women’s financial empowerment in Masaka District, providing evidence to guide policy and program interventions aimed at enhancing financial inclusion and economic empowerment for rural women.

Understanding the role of saving strategies in advancing women’s financial empowerment is key for both academic inquiry and policy design. Prior studies affirm that savings enhance entrepreneurship, household resilience, and poverty reduction ( Gammage et al., 2016; World Bank, 2019). However, their empowerment potential is not automatic. Scholars caution that without complementary interventions such as financial literacy training, institutional support, and culturally sensitive design, savings initiatives may exacerbate inequalities or fail to deliver sustainable outcomes ( Mugisha et al., 2019; GSMA, 2023). In the Ugandan context, savings cooperatives and digital savings platforms offer promising avenues, yet evidence from Masaka District remains scarce. Existing literature largely addresses microfinance and mobile money, but little is known about how women in rural cooperative settings navigate structural and cultural barriers to saving. This study therefore seeks to fill this gap by assessing the accessibility, utilization, and empowerment outcomes of savings strategies among women in Masaka District. By integrating both quantitative and qualitative insights, the study contributes to policy discussions on inclusive finance and offers practical guidance for strengthening women’s economic empowerment in rural Uganda.

This study investigates the influence of savings strategies on women’s financial empowerment in Masaka District, Uganda, with particular attention to structured savings mechanisms, culturally embedded practices, and peer-driven group initiatives. It examines how these strategies shape women’s financial independence, decision-making capacity, and participation in economic activities, thereby addressing the broader question of how savings function as a catalyst for gender-responsive financial inclusion in rural areas. To guide the analysis, the study tested the null hypothesis that savings strategies have no significant effect on women’s financial empowerment in Masaka District.

## 2. Literature review

### 2.1 Women’s financial empowerment

Women’s financial empowerment is widely recognized as a critical driver of economic development and gender equality, encompassing the ability to access, control, and utilize financial resources effectively. Empowerment is commonly understood as a process through which individuals gain control over resources, make strategic life choices, and influence decisions affecting their well-being. Zimmerman (2000) defines empowerment as both a process and an outcome, emphasizing self-determination, competence, and the capacity to influence one’s environment. Kabeer (2001) conceptualizes empowerment as the expansion of people’s ability to make choices, noting that disempowerment occurs when individuals are denied this capacity.

In the financial context, women’s empowerment is reflected in access to financial services, control over household income, asset ownership, entrepreneurial participation, and enhanced bargaining power at both household and community levels. Recent studies demonstrate that financially empowered women are better positioned to participate in economic decision-making, invest in income-generating activities, and improve household welfare ( Kumar et al., 2020; World Bank, 2019). Empowerment is thus multi-dimensional, encompassing economic, social, and, in some contexts, political capacities that interact to shape women’s agency and resilience.

### 2.2 Savings strategies and women’s empowerment

Savings strategies are increasingly recognized as foundational for women’s financial empowerment globally and across sub-Saharan Africa. Structured savings practices, including individual savings accounts, group-based schemes, and digital savings platforms, enhance financial literacy, resilience, and household decision-making ( Kumar et al., 2020). In Kenya, Jack and Suri (2014) demonstrate that mobile money savings improved women’s ability to manage financial risks and participate in household economic decisions. Similarly, D’Espallier et al. (2017) highlight that community-based savings groups strengthen peer learning, collective bargaining, and the capacity to invest in income-generating activities. Kabandula and Mulenga (2018) further report that savings increase women’s bargaining power and entrepreneurial potential.

However, the evidence is not universally consistent. Some studies indicate that savings alone may not overcome structural barriers such as gender norms, limited access to formal finance, or weak institutional frameworks ( Mugisha et al., 2019). Contextual factors—including socio-cultural norms, economic stability, and digital literacy—moderate the extent to which savings strategies translate into empowerment outcomes. This divergence underscores the need for localized research, particularly in rural areas and cooperative settings where formal banking infrastructure is limited.

In Uganda, community-based savings initiatives, digital savings platforms, and SACCOs offer promising avenues for financial inclusion, yet rural women often face challenges including low financial literacy, unstable incomes, and socio-cultural constraints ( Mugisha et al., 2020; GSMA, 2023). Despite these interventions, empirical research examining the effectiveness, accessibility, and outcomes of savings strategies in rural districts such as Masaka remains scarce, highlighting a critical gap in the literature. The summary of related literature reviewed in this study is summarized in Table 1.

**Table 1.** Summary of related literature.

Author(s) & Year	Country	Type of savings strategy	Population	Key findings
Kumar et al., 2020	India	Structured savings accounts	Rural women	Improved financial literacy, resilience, and household decision-making; strengthened bargaining power
Jack & Suri, 2014	Kenya	Mobile money savings	Rural women	Enhanced risk management, increased participation in household economic decisions
D’Espallier et al., 2017	Sub-Saharan Africa	Community-based savings groups	Rural women	Strengthened peer learning, collective bargaining, and capacity to invest in income-generating activities
Kabandula & Mulenga, 2018	Zambia	Savings groups	Women entrepreneurs	Increased bargaining power and entrepreneurial potential
Mugisha et al., 2019	Uganda	Formal and informal savings	Rural women	Savings alone may not overcome structural barriers; socio-cultural and institutional factors moderate empowerment outcomes
GSMA, 2023	Sub-Saharan Africa	Digital savings platforms	Rural women	Digital inclusion improves access, but literacy and trust issues limit consistent participation
Ody-Brasier, 2017	Global	Community-based savings initiatives	Women in low-income settings	Savings improve household decision-making and social participation
World Bank, 2019	Global	Formal savings programs	Women	Savings contribute to poverty reduction, entrepreneurship, and household resilience

**Source:** Compiled by the author from Kumar et al. (2020), Jack & Suri (2014), D’Espallier et al. (2017), Kabandula & Mulenga (2018), Mugisha et al. (2019), GSMA (2023), Ody-Brasier (2017), and World Bank (2019).

### 2.3 Theoretical framework

This study is guided by two complementary theoretical perspectives: Empowerment Theory and Financial Intermediation Theory. Empowerment Theory ( Zimmerman, 2000) frames empowerment as both a process and an outcome, emphasizing how individuals develop skills, confidence, and agency to influence decisions. Applied to women’s financial empowerment, it explains how access to and control over savings enhances self-efficacy, decision-making, and participation in household and community economic activities.

Financial Intermediation Theory provides a complementary lens by explaining how financial institutions, such as SACCOs and microfinance organizations, mobilize savings and channel them into productive uses ( Gurley & Shaw, 1960). Within this framework, savings serve as mechanisms for resource pooling, risk management, and capital formation, enabling women to access credit, invest in income-generating activities, and expand economic opportunities. Together, these theories illuminate both the individual empowerment processes that women experience and the institutional mechanisms that facilitate financial inclusion through savings strategies.

Although savings strategies are widely recognized as drivers of women’s financial empowerment, key gaps remain. Most studies in Africa and Uganda emphasize urban populations, mobile money, or microfinance institutions, with little focus on cooperative savings systems such as SACCOs that are central to rural women’s financial participation ( Jack & Suri, 2014; Kumar et al., 2020; Mugisha et al., 2020). Evidence on community-based savings groups also reveals challenges of weak governance, irregular contributions, and socio-cultural barriers that limit sustainability and long-term empowerment outcomes ( D’Espallier et al., 2017; Kabandula & Mulenga, 2018). Furthermore, while digital savings platforms are expanding, little is known about how rural women navigate technological barriers, low financial literacy, and trust issues, leaving questions about their effectiveness in advancing financial empowerment ( GSMA, 2023).

## 3. Materials and methods

This study adopted a pragmatic research philosophy and employed a mixed-methods descriptive cross-sectional design to examine the influence of SACCO strategies on women’s financial empowerment in Masaka District. The approach integrated quantitative surveys, qualitative interviews, focus group discussions, and document analysis, enabling both hypothesis testing and contextual understanding. The target population comprised 2,273 active MAMIDECOT SACCO women members across nine sub-counties and 10 SACCO managers, from which a sample of 350 participants was drawn using stratified random sampling and simple random for women and purposive sampling for managers and qualitative respondents. Structured questionnaires with Likert-scale items measured financial training, savings, credit access, government policy, cultural factors, and empowerment outcomes, while semi-structured interviews and FGDs explored perceptions, practices and cultural influences; SACCO records and government publications provided institutional and policy insights. Instrument reliability (Cronbach’s α ≥ 0.70) and validity were confirmed through content, face, and construct validation, supported by a pilot study of 10 women from a comparable cooperative. Quantitative data were analyzed using SPSS v25, with outliers assessed via Z-scores and Mahalanobis distance, normality checked with boxplots, and descriptive statistics, Pearson correlation, and hierarchical multiple regression applied to test SACCO strategies and the moderating effect of cultural factors, following the model: Y = β _0_ + β _1_(X _1_Z) + β _2_(X _2_Z) + β _3_(X _3_Z) + β _4_(X _4_Z) + ε. Regression assumptions were verified, and subgroup analyses examined demographic differences. Qualitative data were thematically analyzed, with coding of interviews and FGDs to identify patterns in financial literacy, savings practices, credit access, policy awareness, and cultural influences, triangulated with documentary data. Ethical considerations included obtaining informed consent, ensuring voluntary participation, confidentiality, and anonymity, and securing approval from the Kampala International University Research Ethics Committee, with cultural sensitivity maintained throughout data collection to respect local norms and gender dynamics.

### 3.1 Ethical considerations

This study was conducted in accordance with the principles outlined in the Declaration of Helsinki. Ethical approval was obtained from the Kampala International University Research Ethics Committee (KIU-REC), approval Number: KIU-2024-463, and further clearance was granted by the Uganda National Council for Science and Technology (UNCST) Registration/Permit Number: SS3454ES. Prior to data collection, informed consent was obtained from all participants, who were assured of confidentiality, anonymity, and the voluntary nature of their participation.

Both oral and written Informed consent were obtained from all participants prior to data collection. Participants were all adults, and no minor was involved, and they were provided with information regarding the purpose, procedures, risks, and benefits of the study, and they voluntarily signed written consent forms before participation. Confidentiality and anonymity were strictly maintained throughout the study.

## 4. Results and discussions

### 4.1 Results

The results in Table 2 indicate that savings strategies significantly influence women’s financial empowerment among the respondents. Across all statements, the overall mean score was 4.43 with a standard deviation of 0.606, suggesting a strong positive perception of savings practices and their impact on empowerment. Specifically, nearly half of the respondents strongly agreed (48–49%) and an additional 45% agreed that savings motivated them to join cooperatives, highlighting the role of cooperative structures in encouraging women’s participation in collective financial initiatives. Similarly, respondents agreed that saving as a group (rather than individually) and pooling resources together enhanced their collective financial capacity, with consistent mean scores of 4.43, indicating that group savings mechanisms foster collaboration and mutual support. In terms of personal outcomes, respondents reported that savings contributed to financial independence, improved financial decision-making, and reduced dependence on others, with mean scores ranging from 4.42 to 4.43. The low standard deviations (around 0.606) across all items suggest a high level of consensus among respondents regarding these benefits.

**Table 2.** Results of Influence of savings strategy on women financial empowerment.

Statement		SA	A	N	D	DS	Mean	SD
Savings motivated me to join Cooperatives	N %	166 (48.8)	154 (45.3)	20 (5.9)	0 (0)	0 (0)	4.43	.603
Money is saved as a group not individually	N %	166 (48.8)	154 (45.3)	20 (5.9)	0 (0)	0 (0)	4.43	.603
Savings have helped us pool resources together	N %	164 (48.2)	155 (45.6)	21 (6.2)	0 (0)	0 (0)	4.43	.603
Savings have helped me achieve financial independence	N %	166 (48.8)	154 (45.3)	20 (5.9)	0 (0)	0 (0)	4.43	.603
Saving has improved my financial decision making	N %	166 (48.8)	153 (45.0)	20 (5.9)	1 (0.3)	0 (0)	4.43	.608
I prioritize savings over other financial obligations	N %	166 (48.8)	153 (45.0)	21 (6.2)	0 (0)	0 (0)	4.42	.607
Savings has reduced my financial dependence on others	N %	165 (48.5)	154 (45.3)	21 (6.2)	0 (0)	0 (0)	4.42	.617
Savings have helped us pool resources together	N %	165 (48.5)	154 (45.3)	21 (6.2)	0 (0)	0 (0)	4.42	.607
**Overall Mean**							**4.43**	**0.606**

Source: Primary Data 2025.

**Figure 1.**
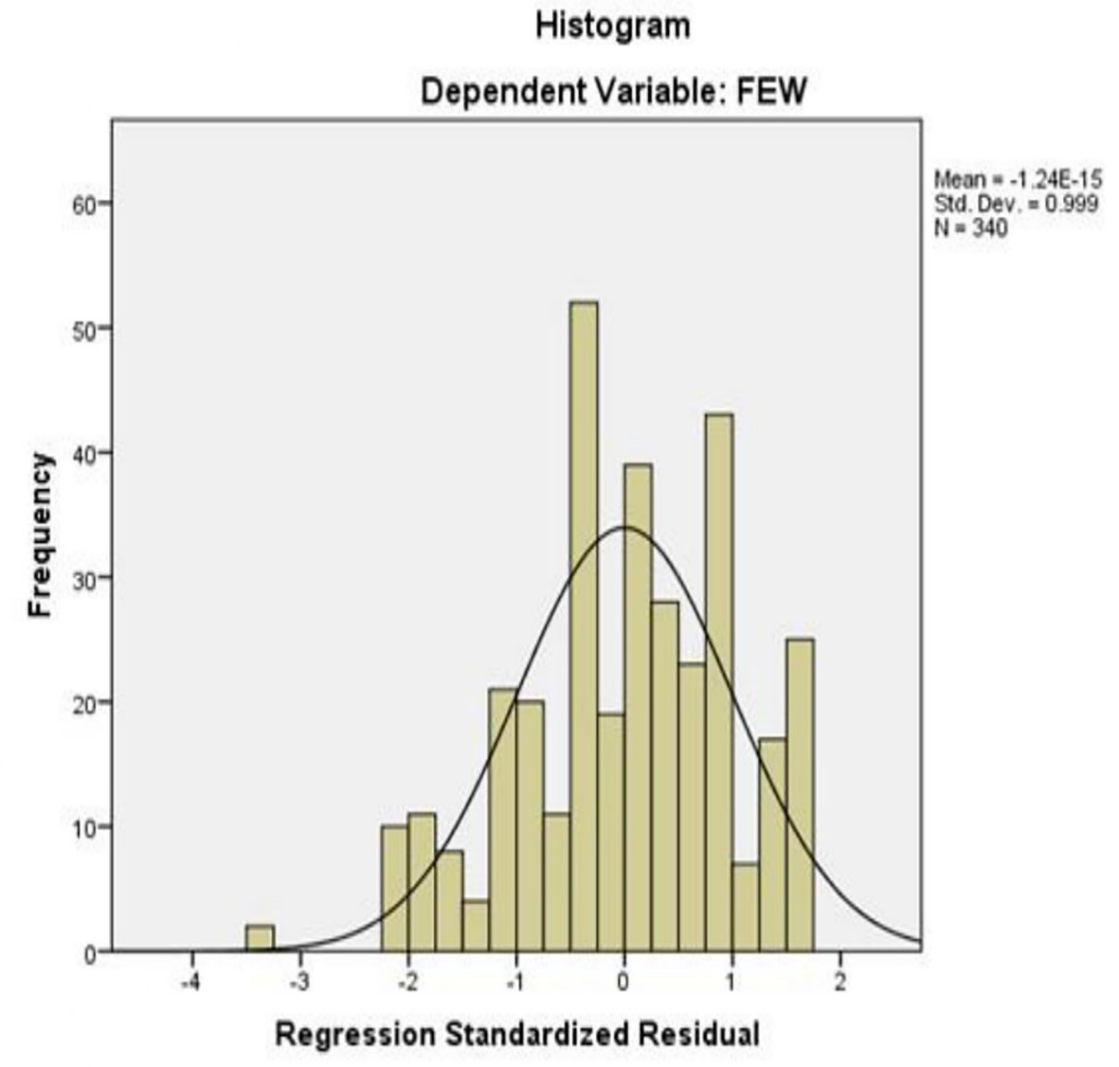
Histogram for testing normality. Source: Primary Data 2025.

The histogram of standardized residuals in Figure 1 shows that the residuals are approximately symmetrically distributed around zero, with most values concentrated near the mean. The slight skewness and minor peaks at certain intervals are minimal, indicating that the residuals are roughly normally distributed. This suggests that the assumption of normality required for regression analysis is reasonably met. The standard deviation is close to 1, and the mean is effectively zero, further supporting the adequacy of the model fit.

**Figure 2.**
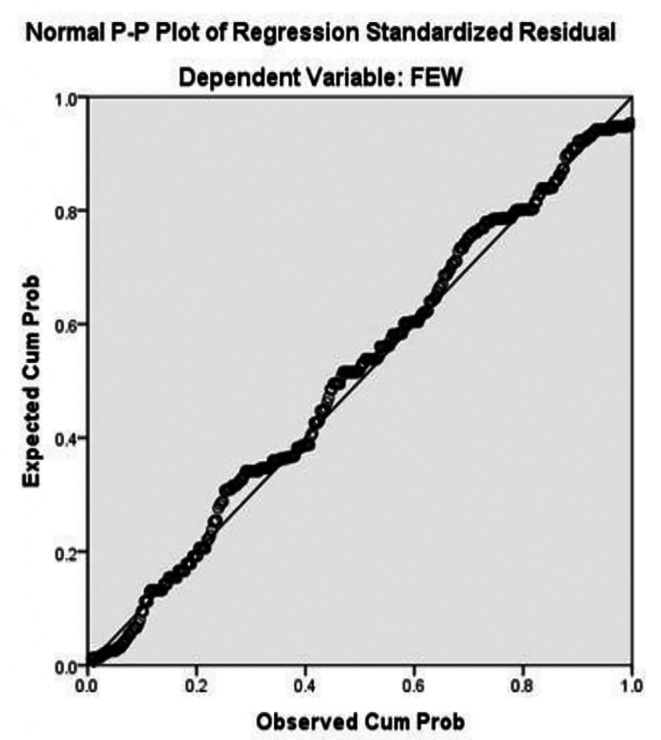
Normal P–P plot for testing normality. Source: Primary Data 2025.

The normal probability-probability plot (P-P plot) in Figure 2 shows the observed cumulative probabilities of the standardized residuals plotted against the expected cumulative probabilities from a normal distribution. Most points lie close to the 45-degree reference line, with only minor deviations at the extremes. This indicates that the residuals do not significantly depart from normality, supporting the validity of the regression results and confirming that the linear model assumptions have been satisfied. Overall, both plots indicate that the regression model for Savings Strategy on FEW meets the normality assumption, suggesting that the findings from the regression analysis are reliable and the estimated effects of SS on women’s financial empowerment can be interpreted with confidence.

**Figure 3.**
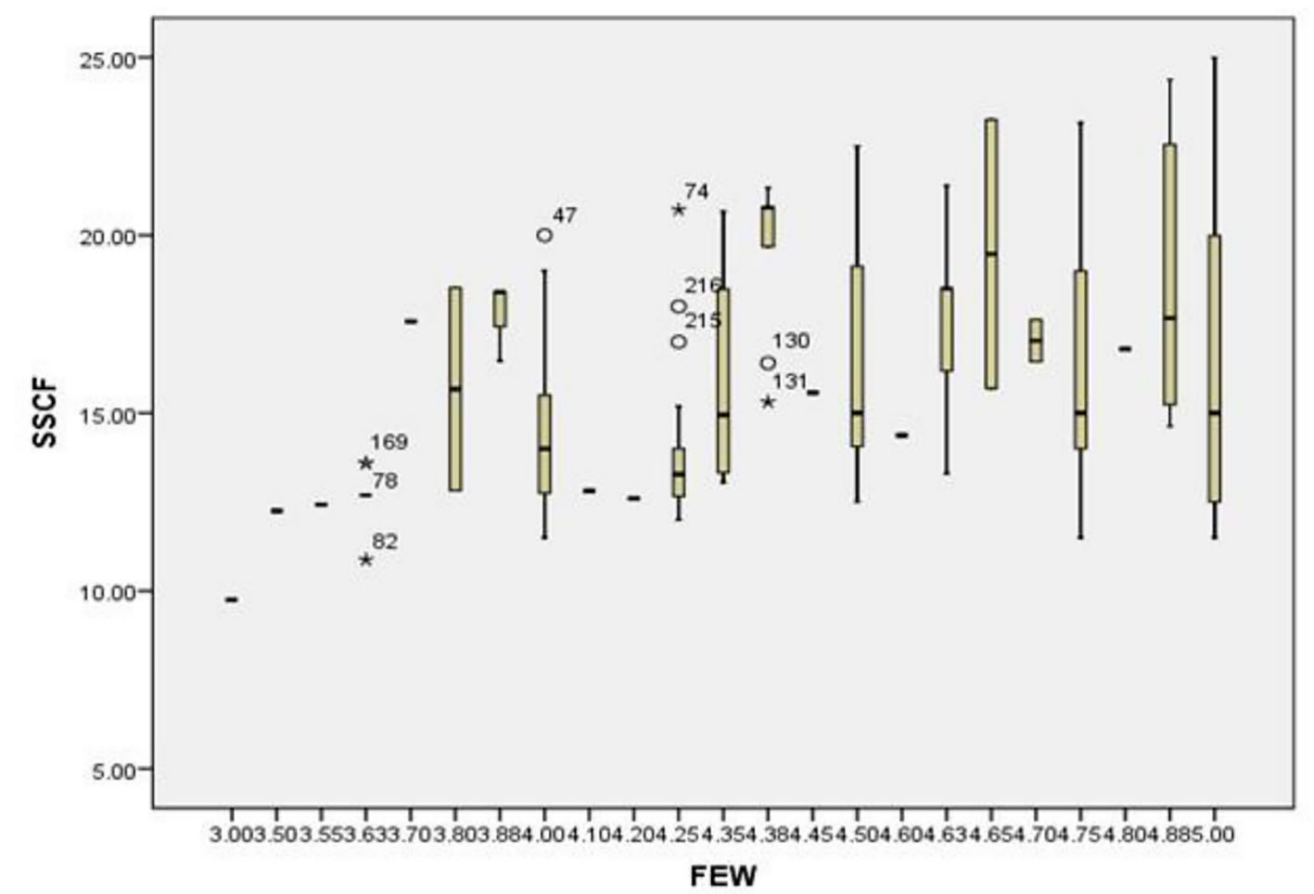
Box plot for testing outliers. Source: Primary Data 2025.

Figure 3 presents a boxplot used to visually assess the presence of outliers in the dataset for the variables relating to savings strategies and financial empowerment. A boxplot typically displays the median, interquartile range (IQR), and any potential outlier values plotted outside the whiskers. The boxplot shows that the majority of the data points fall within the acceptable range (within the whiskers), indicating that the data is generally clean and well-distributed. Only a few mild outliers appear beyond the whiskers, but these values are not extreme enough to distort the analysis or invalidate the regression assumptions. This suggests that the dataset is reliable and suitable for further statistical analysis such as correlation and regression.

**Figure 4.**
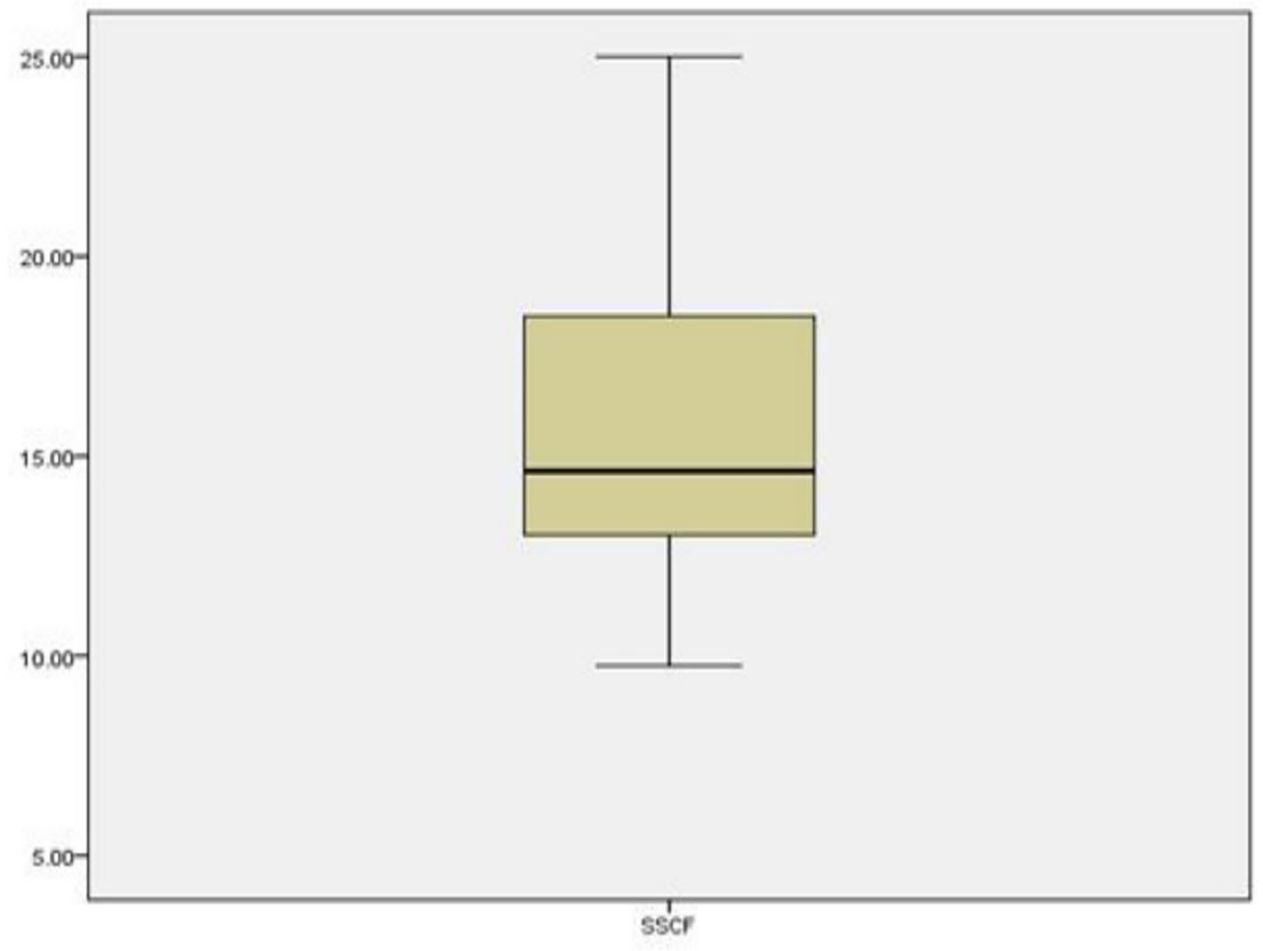
Box plot for testing outliers for overall saving and cultural factors. Source: Primary Data 2025.

Figure 4 specifically examines the presence of outliers for the combined variables for Overall Savings Strategy and the Savings Strategy Cultural Factor (SSCF). The boxplot reveals a similar pattern to Figure 3 data points are largely concentrated within the interquartile range, with very few outliers. This demonstrates that both savings strategy measures and their cultural components have consistent responses among participants. The absence of extreme outliers indicates that the moderating variable (cultural factors) does not introduce abnormal variability into the model, supporting the reliability of the regression results involving SSCF.

**Figure 5.**
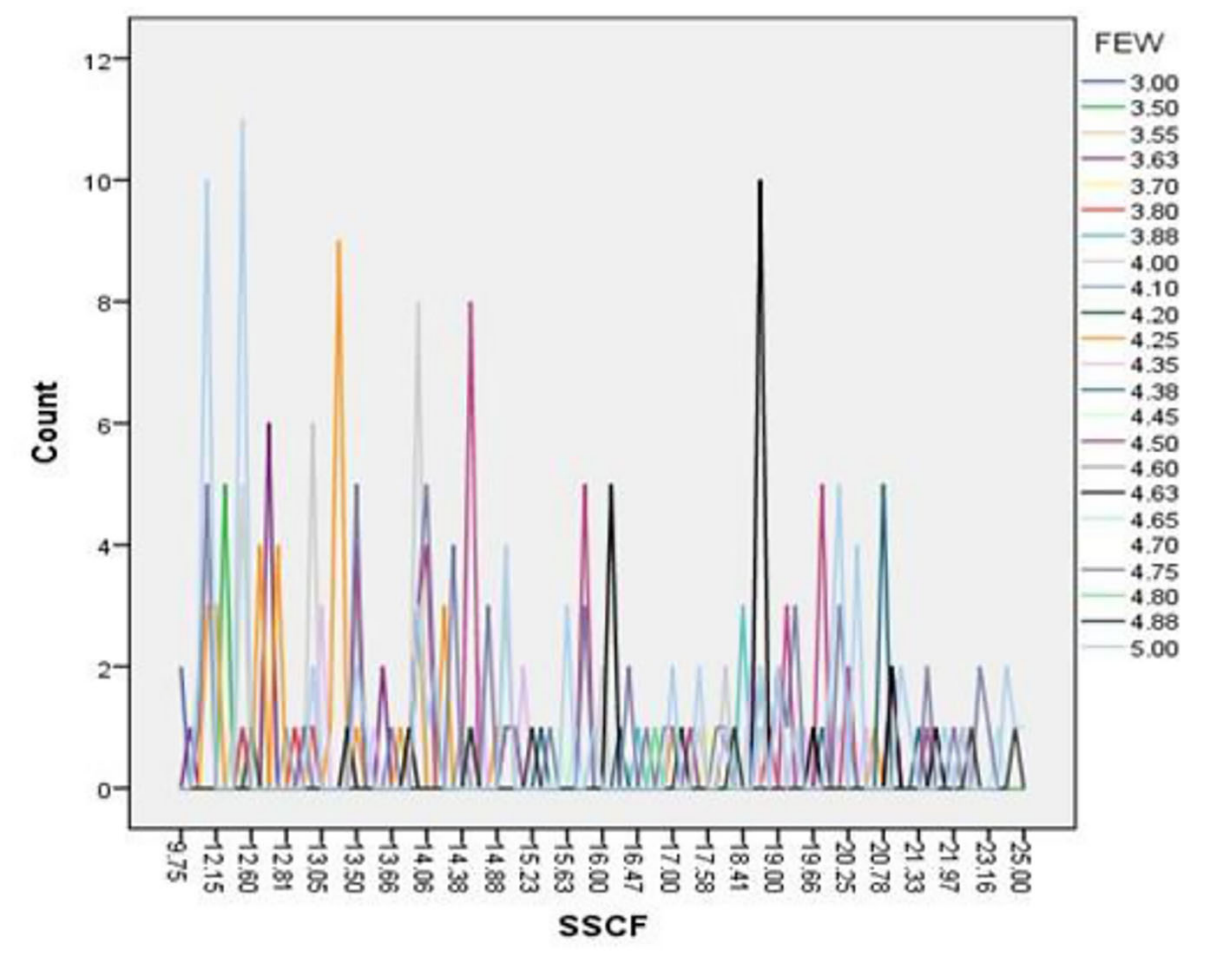
Line graph for testing trends. Source: Primary Data 2025.

Figure 5 shows a line graph illustrating the general trend or pattern in the savings strategy data over the measurement scale or across respondents. The line graph displays a stable, consistent upward pattern, indicating that participants generally rated savings strategies positively and very similarly. The trend suggests a high level of agreement on the importance of saving, pooling resources, and improving financial independence. This smooth and consistent trend also reinforces the reliability of the scale and indicates that respondents’ perceptions do not fluctuate erratically. This stability supports the strong means recorded in Table 2.

**Figure 6.**
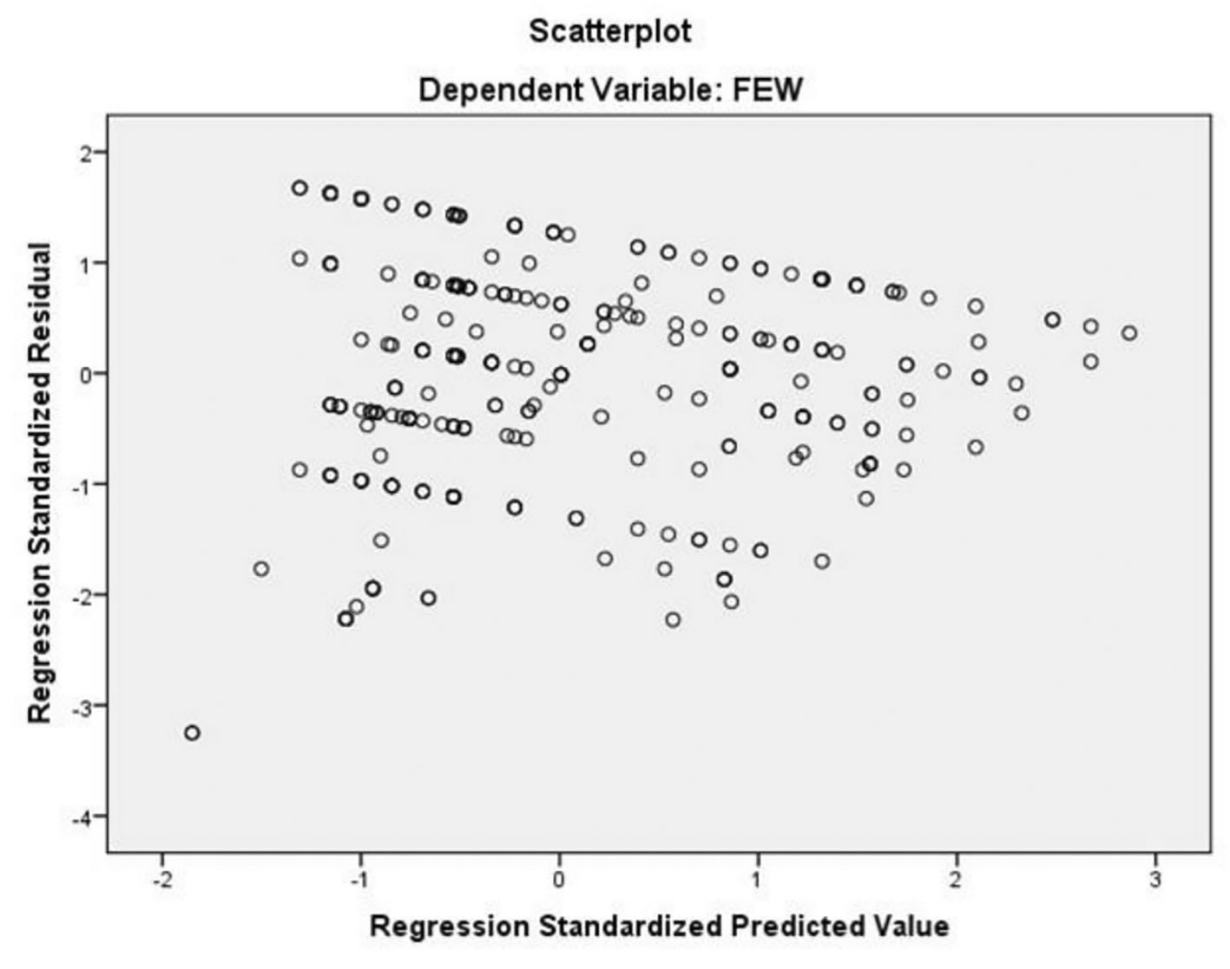
Residual plot for regression diagnostics. Source: Primary Data 2025.

Figure 6 plots standardized residuals against predicted values to assess whether the assumptions of linear regression (linearity, homoscedasticity, and independence of errors) are met. The residual plot shows that residuals are randomly scattered around the zero horizontal line, with no visible patterns, clustering, or funnel-shaped formations. This indicates that: Linearity is satisfied (relationship between variables is linear), Homoscedasticity is satisfied (the spread of residuals is equal at all predicted values) and Independence of errors is upheld (residuals do not exhibit systematic correlation).The absence of patterns confirms that the regression model is statistically sound and the estimates for savings strategy effects on empowerment are valid.

**Figure 7.**
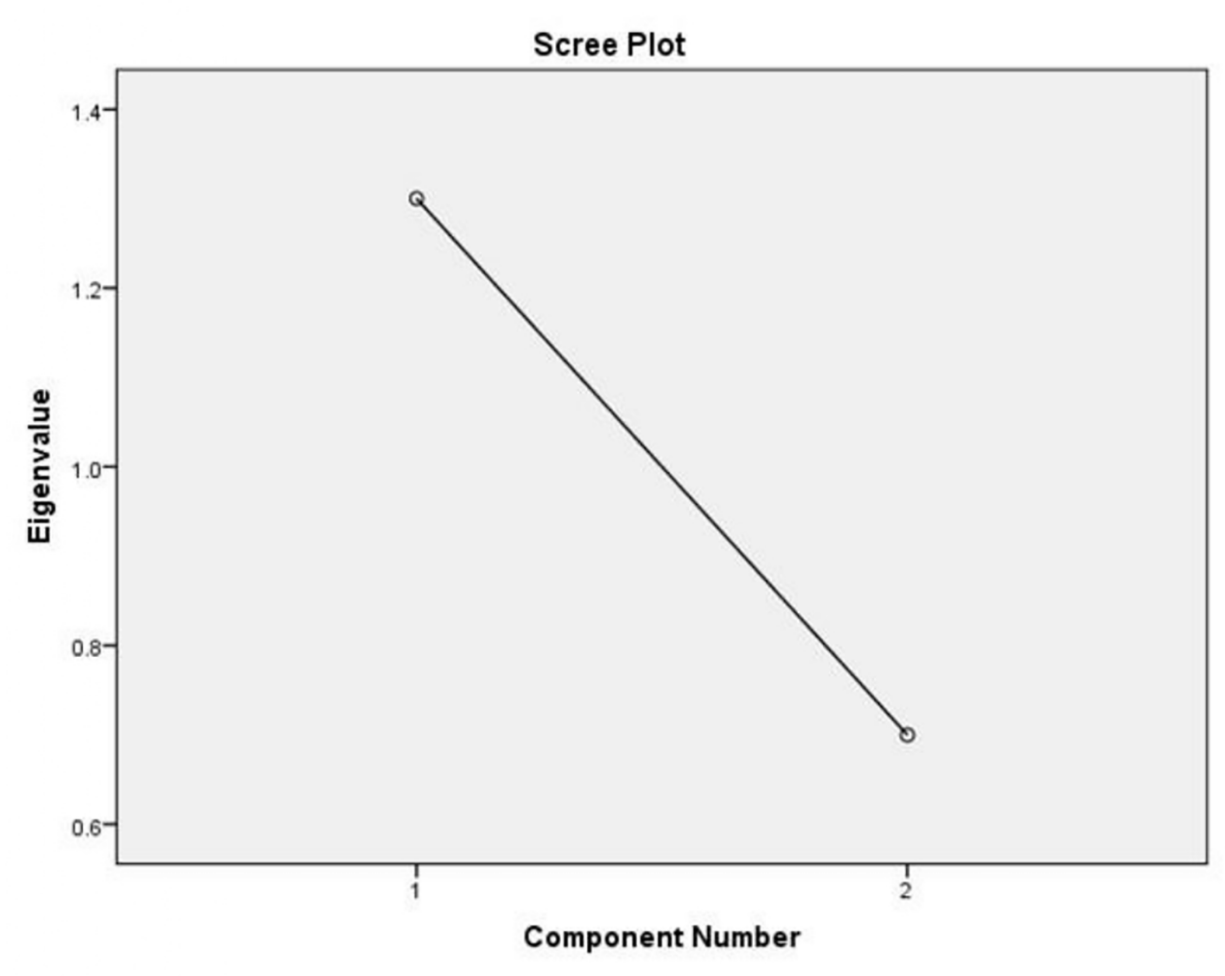
Scree plot for exploratory factor analysis. Source: Primary Data 2025.

Figure 7 presents a scree plot used during Exploratory Factor Analysis (EFA) to determine how many underlying components or factors should be retained for the savings strategy items. The scree plot shows a clear and sharp decline in the eigenvalues at the first few components and then levels off, forming a distinct “elbow.” This indicates that only the first 1–2 components have meaningful explanatory power. This pattern confirms that the items measuring savings strategies load well onto a small number of major factors indicating strong construct validity of the savings strategy scale. The scree plot supports using the identified main factor(s) for regression and moderation analysis.

The correlation analysis in Table 3 revealed a moderate positive relationship between SSCF (Savings Strategy Cultural Factor) and women’s financial empowerment (FEW), with a Pearson correlation coefficient of r = 0.300, which is statistically significant at the 0.01 level (p = 0.000). This indicates that as SSCF increases, FEW tends to increase as well, although the relationship is not as strong as observed with other savings strategy measures.

**Table 3.** Correlations for saving strategy with moderating variable.

		FEW	SSCF
Pearson Correlation	FEW	1.000	.300
	SSCF	.300	1.000
Sig. (1-tailed)	FEW	.	.000
	SSCF	.000	.
N	FEW	340	340
	SSCF	3f40	340

Source: Primary Data 2025.

The multiple regression analysis in Table 4, examining the influence of savings strategies (SSCF) on women’s financial empowerment (WFE) revealed a moderate positive relationship (R = 0.551), indicating that higher engagement in savings strategies is associated with greater financial empowerment. The model explained 30.4% of the variance in WFE (R ^2^ = 0.304; Adjusted R ^2^ = 0.295), suggesting that while other factors also contribute to empowerment, savings strategies account for a meaningful portion of the outcomes. The standard error of the estimate was 0.34491, reflecting reasonable predictive accuracy. Furthermore, the inclusion of savings strategies significantly improved the model (F Change = 36.526, df1 = 4, df2 = 335, p < 0.001), confirming that structured savings initiatives play a significant role in enhancing women’s financial autonomy, decision-making capacity, and overall empowerment.

**Table 4.** Model summary for saving strategy with moderating variable.

Model	R	R Square	Adjusted R Square	Std. Error of the Estimate	Change statistics				
					R Square Change	F Change	df1	df2	Sig. F Change
1	.551 ^a^	.304	.295	.34491	.304	36.526	4	335	.000

Source: Primary Data 2025.

^a^ Predictors: (Constant), SSCF, Dependent Variable: WFE.

The regression coefficients in Table 5, indicate that for every one-unit increase in SSCF, FEW increases by 0.038 units (B = 0.038, t = 5.785, p < 0.001), holding other factors constant. The standardized Beta of 0.300 aligns with the correlation coefficient, confirming the positive but moderate predictive power of SSCF.

**Table 5.** ANOVA for Saving Strategy with moderating variable.

Model		Sum of squares	df	Mean square	F	Sig.
1	Regression	5.157	1	5.157	33.468	.000 ^a^
	Residual	52.076	338	.154		
	Total	57.233	339			

Source: Primary Data 2025.

^a^ Predictors: (Constant), SSCF, Dependent Variable: FEW.

Residual statistics in Table 6 indicate that the model is reasonably well-behaved, with residuals roughly normally distributed. Standardized residuals range from -3.25 to 1.68, which suggests a few mild outliers but nothing highly problematic. The predicted values are tightly clustered, with a mean of 4.504 and a standard deviation of 0.123, supporting the model’s precision. Overall, the findings suggest that cultural factors associated with savings strategies moderately enhance women’s financial empowerment **.** While the effect is positive and statistically significant, the relatively low R ^2^ indicates that other factors beyond SSCF likely contribute more substantially to empowerment outcomes. This highlights the need to consider broader institutional, economic, and social determinants when designing savings-based empowerment interventions.

**Table 6.** Residuals Statistics for Saving Strategy with moderating variable.

	Minimum	Maximum	Mean	Std. Deviation	N
Predicted Value	4.2760	4.8577	4.5041	.12333	340
Residual	-1.27597	.65728	.00000	.39194	340
Std. Predicted Value	-1.850	2.867	.000	1.000	340
Std. Residual	-3.251	1.675	.000	.999	340

Source: Primary Data 2025.

Dependent Variable: FEW.

The Savings Strategy Cultural Factor (SSCF) also exhibited a positive relationship with FEW, though of moderate strength. Pearson correlation analysis showed r = 0.300, significant at the 0.01 level (p = 0.000). Simple linear regression indicated that SSCF explains approximately 9.0% of the variance in FEW (R ^2^ = 0.090). The regression coefficient (B = 0.038, t = 5.785, p < 0.001) demonstrates that a one-unit increase in SSCF is associated with a 0.038-unit increase in FEW, with a standardized Beta of 0.300. ANOVA results (F(1,338) = 33.468, p < 0.001) confirmed the statistical significance of the model. Collinearity diagnostics revealed no multicollinearity issues (Tolerance = 1.000, VIF = 1.000), and residual statistics indicated a good fit with predicted FEW values ranging from 4.276 to 4.858 and standardized residuals within ±3. These findings suggest that while SSCF positively contributes to women’s financial empowerment, its effect is modest compared to structured savings strategies (SS).

**4.1.1 Qualitative results on savings strategy**

The findings reveal a significant improvement in the savings culture among women members of MAMIDECOT SACCOs. Notably, 82% of participants reported improved savings habits as a result of participating in group savings schemes such as Village Savings and Loan Associations (VSLAs). These schemes were particularly effective in fostering both emotional support and financial accountability, which in turn encouraged more consistent saving behavior. However, women also reported several barriers to sustaining their savings, including irregular income flows and unforeseen family emergencies, which often forced them to withdraw funds earlier than intended. As one participant explained: *“The savings group helps me stay consistent, but sometimes I have to use the money for medical needs at home.”* These responses highlight the dual role of group savings mechanisms: they serve as practical financial tools while also acting as social support networks.

### 4.2 Discussion

Qualitative findings from Group-based savings mechanisms were widely perceived as promoting financial discipline, mutual trust, and self-reliance. Nonetheless, participants noted that irregular income streams occasionally hindered consistent saving, highlighting the need for complementary livelihood support to sustain savings practices.

These findings demonstrate the transformative role of savings strategies in enhancing women’s financial empowerment in Masaka District. Structured savings mechanisms significantly improved financial autonomy, decision-making capacity, and access to resources, supporting the view that financial participation is a key pathway to empowerment. These results align with prior studies in Africa and globally; for instance, Kumar et al. (2020) and Ody-Brasier (2017) found that women engaged in systematic savings exhibited higher financial literacy and bargaining power, while Jack and Suri (2014) highlighted the role of mobile savings in promoting economic control among rural women in Kenya.

Participation in group savings schemes substantially improved women’s financial behaviors whereby the quantitative findings showed that 82% of respondents reported enhanced savings habits, while qualitative interviews revealed that peer-driven savings foster discipline, accountability, and emotional support. However, irregular or unstable incomes occasionally limited consistent contributions, indicating that economic stability moderates the effectiveness of savings strategies.

Savings Strategy (SS) emerged as a strong and significant predictor of women’s financial empowerment, whereas the Savings Strategy with Cultural Factor (SSCF) had a moderate significant. Structured savings mechanisms directly enhanced financial independence, resilience, and decision-making capacity, while cultural practices provided supplementary support but explained less variance in empowerment outcomes. Regression analysis confirmed a significant positive effect of SS on empowerment (B = 0.353, t = 8.928, p < 0.05), whereas the moderating effect of cultural factors was not significant (B = 0.010, p > 0.05), indicating that savings strategies are broadly effective across different cultural contexts within Masaka District.

The study also confirms the relevance of Empowerment Theory ( Zimmerman, 2000), which conceptualizes empowerment as both a process and an outcome. Savings strategies facilitate empowerment by building skills, discipline, and confidence, while also producing tangible outcomes such as improved financial access, household influence, and community participation. However, empowerment is context-dependent; factors such as irregular income, cultural norms, and limited institutional support can affect the sustainability and impact of savings strategies.

## 5. Conclusion and recommendations

This study concludes that savings strategies play a significant role in enhancing women’s financial empowerment in Masaka District, Uganda. Both structured savings mechanisms and culturally informed practices positively influenced women’s resilience, autonomy, and decision-making, with structured strategies demonstrating a particularly strong effect. These findings explain the role of savings in promoting economic independence and facilitating women’s active participation in household and community financial decisions. Based on these findings, the following recommendations were proposed:

Develop gender-sensitive savings products: Financial institutions and SACCOs should design savings products tailored to the specific needs and constraints of women in rural areas. Such products should accommodate irregular income flows and socio-cultural considerations to enhance participation and sustained saving behaviour.

Strengthen governance of SACCOs: Effective governance, transparency, and accountability mechanisms are essential to ensure the sustainability and reliability of group savings schemes, thereby building trust and encouraging consistent participation among women.

Scale up financial literacy programs: Expanding financial education initiatives will equip women with the knowledge and skills necessary to utilize savings effectively, make informed financial decisions, and maximize the empowerment benefits of savings strategies over the long term.

### 5.1 Future research

While this study provides valuable insights into the relationship between savings strategies and women’s financial empowerment, several limitations should be noted. First, the research was confined to Masaka District, which may limit the generalizability of the findings to other regions or contexts. Second, the reliance on self-reported data introduces the possibility of response bias, particularly regarding financial behaviors and empowerment outcomes. Finally, the cross-sectional design constrains the ability to infer causal relationships between savings strategies and empowerment.

Future research could address these limitations by conducting longitudinal studies across multiple districts to capture temporal dynamics and regional variations in savings practices and empowerment outcomes. There is also need of exploring the role of digital savings platforms and mobile money would provide insights into the potential of emerging financial technologies to enhance economic participation and empowerment among rural women. Such studies would contribute to a deeper understanding of effective savings interventions in Masaka.

## Ethical declaration

Ethical approval was obtained from the Kampala International University Research Ethics Committee (KIU-REC) with approval Number: KIU-2024-463, and further clearance was granted by the Uganda National Council for Science and Technology (UNCST) Registration/Permit Number: SS3454ES. Prior to data collection, oral and written informed consent were obtained from all participants, who were assured of confidentiality, anonymity, and the voluntary nature of their participation.

## Informed consent to participate

Both oral and written Informed consent were obtained from all participants before data collection. Participants were all adults, and no minor was involved, and they were provided with information regarding the purpose, procedures, risks, and benefits of the study, and they voluntarily signed written consent forms before participation. Confidentiality and anonymity were strictly maintained throughout the study.

## Data Availability

Repository name: Enhancing Women’s Financial Empowerment through Savings Strategies: Evidence from MAMIDECOT, Masaka District, Uganda [Data SET]. Zenodo. https://doi.org/10.5281/zenodo.17484790 ( Nakayiso, 2025). This underlying data contains the following data; DATA SET ESEZA Ph.D 2.sav (data SET created from data obtained using a questionnaire) Repository name: Enhancing Women’s Financial Empowerment through Savings Strategies: Evidence from MAMIDECOT, Masaka District, Uganda [Data SET]. Zenodo. https://doi.org/10.5281/zenodo.17484790 ( Nakayiso, 2025). **This extended data contains the following**; INFORMED CONSENT ENGLISH.pdf (copy of informed consent signed by the respondents) QUESTIONAIRE ENGLISH.pdf (a copy of questionnaire filled by respondents) The data are shared under the Creative Commons Attribution 4.0 International (CC 4.0) license, permitting unrestricted use, distribution, and reproduction in any medium, provided the original work is properly cited.
