# Supplementary material for: Enhancing Women’s Financial Empowerment through Savings Strategies: Evidence from MAMIDECOT, Masaka District, Uganda
Source: F1000Res. 2025 Nov 25;14:1308. [Version 1] doi: 10.12688/f1000research.171318.1 (PMC12993285; doi:10.12688/f1000research.171318.1)
Supplement: Peer review history

# Reviewer report: Reviewer response for version 1

**DOI:** 10.5256/f1000research.188915.r464004

**Published:** 2026-03-31

**Authors:** Teresa Felgueira

**Affiliations:** 1 Polytechnic Institute of Guarda, Guarda, Portugal

The study investigates how savings strategies influence women's financial empowerment within the MAMIDECOT SACCO in the Masaka District of Uganda. Using a convergent mixed-methods approach, the authors analyzed quantitative data from 340 women and qualitative insights from 10 managers and focus group discussions. The findings suggest that structured savings strategies, including group-based mechanisms and resource pooling, significantly enhance women's financial independence and decision-making capacity. While the study notes that cultural factors did not significantly moderate these effects, it emphasizes the importance of savings as a catalyst for economic participation in rural settings.

 The paper has academic merit and provides valuable insights into the MAMIDECOT SACCO, but the scientific "packaging" of the statistics needs to be corrected to meet the high standards of the publication. While the study addresses a relevant topic and uses a sound methodological framework (Mixed-Methods), it contains technical and reporting inconsistencies that must be addressed before it can be fully "Approved." These issues go beyond "minor changes" because they impact the reader's ability to verify the study's core findings.

 To make the article scientifically sound, the following points must be addressed:

1. **Reconcile Statistical Tables:** Ensure that the R ^2^ value, F-statistic, and degrees of freedom are consistent across the Abstract, the Model Summary, and the ANOVA table.(see the discrepancies between Table 4 and Table 5).
2. **Clarify the Moderation Analysis:** If a moderation model was tested, the authors should present a table showing the interaction effects (e.g., Savings Strategy $\times$ Cultural Factors). Simply reporting "SSCF" as a predictor does not test for moderation.
3. **Provide Full Instrument Details:** Include the complete questionnaire and qualitative interview protocols as an appendix or supplementary file to ensure the study's replicability.
4. **Integrate Qualitative Data:** The "Findings" section mentions qualitative results, but the current text focuses almost entirely on quantitative diagnostic plots (Figures 1–7) . Include direct quotes from focus groups or managers to substantiate the qualitative claims made in the summary.

Thus, to move from "Approved with Reservations" to "Approved," the authors must:

- **Synchronize the data:** Ensure every number in the Abstract matches the tables in the body of the text.
- **Show the "Moderation" math:** If they tested for moderation, they must include the coefficients for the interaction between savings strategies and cultural factors.
- **Provide the Survey Instrument:** Include the questionnaire as supplementary material so other researchers can replicate the study in different districts.

Is the work clearly and accurately presented and does it cite the current literature?

Partly

If applicable, is the statistical analysis and its interpretation appropriate?

Partly

Are all the source data underlying the results available to ensure full reproducibility?

Partly

Is the study design appropriate and is the work technically sound?

Yes

Are the conclusions drawn adequately supported by the results?

Yes

Are sufficient details of methods and analysis provided to allow replication by others?

Yes

Reviewer Expertise:

Marketing; Entrepreneurship; Gender wise; Higher Education; Tourism

I confirm that I have read this submission and believe that I have an appropriate level of expertise to confirm that it is of an acceptable scientific standard, however I have significant reservations, as outlined above.

# Reviewer report: Reviewer response for version 1

**DOI:** 10.5256/f1000research.188915.r464008

**Published:** 2026-03-16

**Authors:** Tahir Iqbal

**Affiliations:** 1 University of Zaragoza, Zaragoza, Aragon, Spain

This study examines the influence of savings strategies on women's financial empowerment among members of MAMIDECOT SACCO in Masaka District, Uganda.

 Using a convergent mixed-methods design with 340 survey respondents and 10 qualitative interviews/focus group discussions, the research finds that savings strategies significantly enhance women's financial independence, decision-making capacity, and household influence (R² = 0.304, p < 0.001).

 The study reports that cultural factors do not significantly moderate this relationship, suggesting structured savings mechanisms are broadly effective across socio-cultural contexts. The authors conclude with recommendations for gender-sensitive savings products, strengthened SACCO governance, and expanded financial literacy programs.

 This manuscript makes a valuable contribution to understanding savings-based women's empowerment in rural Uganda. The mixed-methods design, robust sample, and sound statistical analysis provide credible evidence that savings strategies are associated with enhanced financial empowerment among women in Masaka District. 

 The manuscript make a significant contribution to both the academic literature on women's financial inclusion and the practical evidence base for gender-sensitive financial programming in sub-Saharan Africa.

Is the work clearly and accurately presented and does it cite the current literature?

Yes

If applicable, is the statistical analysis and its interpretation appropriate?

Yes

Are all the source data underlying the results available to ensure full reproducibility?

Yes

Is the study design appropriate and is the work technically sound?

Yes

Are the conclusions drawn adequately supported by the results?

Yes

Are sufficient details of methods and analysis provided to allow replication by others?

Yes

Reviewer Expertise:

NA

I confirm that I have read this submission and believe that I have an appropriate level of expertise to confirm that it is of an acceptable scientific standard.

# Reviewer report: Reviewer response for version 1

**DOI:** 10.5256/f1000research.188915.r436588

**Published:** 2025-12-11

**Authors:** Niyaz Panakaje

**Affiliations:** 1 Yenepoya Research Centre for Finance and Entrepreneurship Development (YEN-REFINED),YIASCM, Yenepoya (Deemed to be University) (Ringgold ID: 219141), Mangaluru, Karnataka, India

**Reviewer report on Enhancing Women’s Financial Empowerment through Savings Strategies: Evidence from MAMIDECOT, Masaka District, Uganda**

**Major Comments**

**Contribution & Significance**

 The study addresses an important issue by examining how savings strategies empower rural women in Uganda, directly linking to Sustainable Development Goals on gender equality and economic inclusion.

 By focusing on the MAMIDECOT cooperative in Masaka District, it fills a gap in localized research and provides empirical evidence of the positive impact of structured savings on women’s financial independence, decision-making, and household influence.

 While the contribution is timely and policy-relevant, it remains largely descriptive. The mechanisms through which empowerment occurs are not fully unpacked, limiting the depth of theoretical and practical insights.

**Methodology**

 The mixed-methods design is appropriate for the research question, combining quantitative surveys with qualitative interviews and focus groups.

 The large sample size strengthens regression analysis, and triangulation across methods adds validity. However, the cross-sectional design prevents causal or long-term conclusions, and reliance on self-reported data introduces social desirability bias.

 The single-district focus restricts generalizability. Further, the reliance on regression analysis is basic.

 A more advanced modeling technique such as Structural Equation Modeling (SEM) could have revealed pathways, mediators, and latent constructs more effectively.

**Theoretical Framing**

 The theoretical framing combines Empowerment Theory and Financial Intermediation Theory, offering a dual-level perspective that is logical and coherent. Empowerment Theory explains empowerment as both process and outcome, while Financial Intermediation Theory highlights the institutional role of SACCOs in mobilizing savings.

 However, this framing is too broad and does not specify which dimensions of empowerment are most affected. It also misses social and cultural mechanisms such as trust, bargaining, and norms. Narrower or alternative frameworks could have provided sharper insights.

 The Capability Approach, Social Capital Theory, Gender and Development frameworks, or Household Bargaining Models would have offered richer explanations of empowerment processes and intra-household dynamics.

**Depth of Analysis**

 The analysis shows that savings strategies had a significant impact, with group savings fostering trust, accountability, and self-reliance. Cultural factors were found to have only a modest moderating effect.

 While these findings are valuable, the study missed opportunities to dig deeper. It did not disaggregate which specific savings practices, such as group trust, pooling, prioritizing, or independence, that drive empowerment. Qualitative data could have been mined more thoroughly to identify mechanisms. SEM could have been used to model latent constructs and indirect effects, offering a more nuanced understanding of how savings strategies translate into empowerment.

**Generalizability**

 The findings are locally valid for Masaka District and may apply to similar rural Ugandan or East African contexts. However, they are less applicable to urban areas or countries with stronger financial infrastructure. The authors themselves acknowledge the need for multi-district and longitudinal studies to strengthen external validity. As it stands, the study provides strong local insights but limited generalizability beyond comparable rural settings.

**Minor Comments**

- The presentation of results is clear, but the discussion could better integrate qualitative findings to highlight mechanisms of empowerment.
- The regression tables and factor analysis are useful, but additional fit indices (if SEM were used) would strengthen methodological rigor.
- The literature review is comprehensive but could be more critically engaged, especially in contrasting contexts where savings strategies fail to empower women.
- Terminology such as “empowerment” could be more precisely defined in economic versus social dimensions to avoid conceptual ambiguity.
- The conclusion is strong but could be expanded to highlight practical implications for policymakers and SACCO managers in more detail.

**Recommendation**

 Overall, the study is a strong starting point but could be improved by narrowing its theoretical lens to economic empowerment or integrating social capital and capability approaches. Methodologically, SEM should be employed to model latent constructs and mediation pathways. Analytically, savings strategies should be disaggregated to identify which practices matter most. The scope should extend beyond Masaka, comparing rural and urban contexts, and longitudinal designs should be used to capture long-term effects. Finally, qualitative depth should be enhanced to probe mechanisms such as trust, discipline, and bargaining more explicitly.

Is the work clearly and accurately presented and does it cite the current literature?

Partly

If applicable, is the statistical analysis and its interpretation appropriate?

Yes

Are all the source data underlying the results available to ensure full reproducibility?

Partly

Is the study design appropriate and is the work technically sound?

Yes

Are the conclusions drawn adequately supported by the results?

Partly

Are sufficient details of methods and analysis provided to allow replication by others?

Yes

Reviewer Expertise:

Developmental Study, Scale and Index Development, Banking and Innovation, SEE

I confirm that I have read this submission and believe that I have an appropriate level of expertise to confirm that it is of an acceptable scientific standard.
